# Comparison of Direct Sequencing, Real-Time PCR-High Resolution Melt (PCR-HRM) and PCR-Restriction Fragment Length Polymorphism (PCR-RFLP) Analysis for Genotyping of Common Thiopurine Intolerant Variant Alleles *NUDT15* c.415C>T and *TPMT* c.719A>G (TPMT*3C)

**DOI:** 10.3390/diagnostics7020027

**Published:** 2017-05-12

**Authors:** Wai-Ying Fong, Chi-Chun Ho, Wing-Tat Poon

**Affiliations:** Department of Clinical Pathology, Pamela Youde Nethersole Eastern Hospital, Chai Wan, Hong Kong Special Administrative Region, China; fwy669@ha.org.hk (W.-Y.F.); hcc604@ha.org.hk (C.-C.H.)

**Keywords:** pharmacogenetic, Asian, genotyping, sequencing, high resolution melt (HRM), Restriction Fragment Length Polymorphism (RFLP), thiopurine intolerance, *NUDT15*, rs116855232, TPMT*3C, rs1142345

## Abstract

Thiopurine intolerance and treatment-related toxicity, such as fatal myelosuppression, is related to non-function genetic variants encoding thiopurine *S*-methyltransferase (TPMT) and Nudix hydrolase 15 (NUDT15). Genetic testing of the common variants *NUDT15*:NM_018283.2:c.415C>T (Arg139Cys, dbSNP rs116855232 T allele) and *TPMT*: NM_000367.4:c.719A>G (TPMT*3C, dbSNP rs1142345 G allele) in East Asians including Chinese can potentially prevent treatment-related complications. Two complementary genotyping approaches, real-time PCR-high resolution melt (PCR-HRM) and PCR-restriction fragment length morphism (PCR-RFLP) analysis were evaluated using conventional PCR and Sanger sequencing genotyping as the gold standard. Sixty patient samples were tested, revealing seven patients (11.7%) heterozygous for *NUDT15* c.415C>T, one patient homozygous for the variant and one patient heterozygous for the TPMT*3C non-function allele. No patient was found to harbor both variants. In total, nine out of 60 (15%) patients tested had genotypic evidence of thiopurine intolerance, which may require dosage adjustment or alternative medication should they be started on azathioprine, mercaptopurine or thioguanine. The two newly developed assays were more efficient and showed complete concordance (60/60, 100%) compared to the Sanger sequencing results. Accurate and cost-effective genotyping assays by real-time PCR-HRM and PCR-RFLP for *NUDT15* c.415C>T and TPMT*3C were successfully developed. Further studies may establish their roles in genotype-informed clinical decision-making in the prevention of morbidity and mortality due to thiopurine intolerance.

## 1. Introduction

Thiopurines, including prodrugs azathioprine, mercaptopurine and thioguanine, are purine analogues that exert their cytotoxic anti-metabolic effect upon conversion to thioguanine nucleotides, causing the inhibition of purine synthesis and base mis-incorporation [1]. Intracellularly, this conversion is limited by the cytosolic enzyme thiopurine *S*-methyltransferase (TPMT), encoded by the *TPMT* gene on chromosome 6, which catalyzes the methylation of thiopurines and metabolites to less active and non-toxic forms. Additionally, the Nudix (Nucleoside Diphosphate linked to X) hydrolase 15, encoded by the *NUDT15* gene on chromosome 13, degrades the thioguanine nucleotides in addition to its native action of elimination of mutagenic 8-oxo-dGTP and exerts an overall protective effect against thiopurine toxicity in vivo [2]. Genetic polymorphisms affecting the expression or turnover of these enzymes therefore affect the pharmacologic response and side effects of thiopurines in patients. 

In 2003, the USA Food and Drug Administration decided to change the drug label to include information about inherited TPMT deficiency [3] and later recommended *TPMT* genotyping or genotypic testing to be performed before initiating treatment with azathioprine [4]. This was due to the finding that individuals homozygous for an inherited defect in the *TPMT* gene displayed increased sensitivity to myelosuppression, the dose-limiting toxicity of the thiopurines [5]. Dose-adjustments (in the case of heterozygotes) or consideration of an alternate agent (in the case of patients homozygous for non-functional alleles) may be required [6] to decrease the risk of potentially fatal metabolite accumulation and myelotoxicity [7]. Among the alleles that are clearly established to be non-functional (*2, *3A, *3B, *3C and *4) [8], the most common are the *3A (allele frequency up to 3.56% in Caucasians) and *3C (allele frequency up to 1.57% in Asians). Of note, as the TPMT*3A allele contains the TPMT*3B c.460G>A and the TPMT*3C c.719A>G variants in cis [9], detection of the *TPMT*:NM_000367.4:c.719A>G (TPMT*3C, dbSNP: rs1142345 G allele) variant alone will also pick up all TPMT*3A variants (which may be further classified by TPMT*3B genotyping/haplotyping).

Polymorphism on the *TPMT* gene, however, could not fully explain the variability in pharmacologic response and myelosuppressive risk of thiopurines [10,11]. In Asian populations, where the allele frequency for non-functional *TPMT* variants (*3A: 0.012%; *3B: 0.0%; *3C: 1.57%; *4–*26: 0.071% [8]) is much lower compared to European [12], Latino [13] and African [14,15] populations, a large-scale genetic association study was performed in a cohort of Korean patients receiving thiopurines to look for additional genetic determinants of thiopurine intolerance [16]. It was revealed that the common missense variant *NUDT15*:NM_018283.2:c.415C>T (Arg139Cys, dbSNP: rs116855232 T allele) conferred susceptibility to thiopurine-induced leukopenia in a genetic dose-dependent manner, with an odds ratio (OR) of 35.6 [16]. The finding has since been replicated in other patient populations, and has confirmed the prognostic value of *NUDT15* c.415C>T genotyping in East Asian populations, where up to more than 20% of the population may be affected and require dose-adjustment or discontinuation [17]. 

As prospective identification of individuals with an exaggerated adverse response to thiopurine can potentially prevent treatment-related complications and improve clinical outcome, the variants TPMT*3C and *NUDT15* c.415C>T were targeted in our patient population, predominantly of Han Chinese (93.6%) [18]. Arguably, an assay with rapid turnaround time is advantageous since it will not delay the sometimes life-saving treatment [19]. On the other hand, to promote adoption of the test, the assay should be simple, objectively interpreted and have low consumable costs compared to traditional sequencing [4], with differentiation based on relative amplification efficiency [20] or by probe-based tests [21,22,23]. Additionally, the assay should be robust to variations caused by pre-analytical variables [24]. With these considerations, two complementary genotyping approaches, real-time PCR-high resolution melt (PCR-HRM) and PCR-restriction fragment length morphism (PCR-RFLP) analysis, were evaluated using conventional PCR and Sanger sequencing genotyping as the gold standard. The two approaches, either alone or in combination, may have the potential to replace existing sequencing-based approaches for genotyping the two pharmacogenetic loci or allow for a more widespread adoption of the test. 

## 2. Materials and Methods 

### 2.1. Clinical Samples and Human Ethics

Archived DNA was retrieved for 60 patients who had been referred for genetic testing at the Clinical Pathology Department, Pamela Youde Nethersole Eastern Hospital. In addition to their original test indications, they additionally gave written informed consent for anonymized testing and assay development using the extracted DNA. The study was reviewed and approved by the Hong Kong Hospital Authority/Hong Kong East Cluster Institutional Review Board Ethics Committee (HKEC-2016-047, 23 August 2016). Non-Chinese patients and patients presenting to the Clinical Pathology Service for *NUDT15* or *TPMT* genetic testing were excluded from this study.

### 2.2. DNA Extraction and Sequencing Genotyping

Human genomic DNA extraction was performed as previously described [25]. Briefly, DNA from peripheral blood was extracted using the QIAamp DNA Blood Mini Kit (Qiagen, Hilden, Germany) following the manufacturer’s instructions and eluted in 100 μL of Tris-EDTA buffer. The extracted genomic DNA was stored at −80 °C until analysis. 

PCR and Sanger sequencing were performed as follows. For *NUDT15* genotyping, each 25 μL reaction contained: 12.5 µL AmpliTaq Gold 360 Master Mix (Applied Biosystems, Foster, CA, USA), 1.0 μL 360 GC Enhancer, 1.0 μM of each of the primers PCP-0023 (5′-CCCAAATAAACACCCTTTGTTTTCTGT-3′) and PCP-0024 (5′-CCTTTGTATCCCACCAGATGGTTC-3′), 20 ng of purified genomic DNA and 7.5 μL of PCR-grade water. Amplification was performed on a Veriti 96-Well Thermal Cycler (Applied Biosystems, Foster, CA, USA) using a stepdown PCR protocol as follows: initial denaturation at 95 °C for 10 min, subsequent denaturation at 95 °C for 30 s, annealing for 30 s with 1 °C decrement per cycle, from 66 to 56 °C for 10 cycles, followed by 30 cycles of annealing at 60 °C for 30 s, extension at 72 °C for 1 min, followed by final extension at 72 °C for 10 min. *TMPT* genotyping PCR was performed using the primers PCP-0027 (5′-CACCCAGCCAATTTTGAGTA-3′) and PCP-0028 (5′-CAGGTAACACATGCTGATTGG-3′), with identical thermo-cycling conditions as above. The PCR products were electrophoresed in 2% agarose in 1× TBE buffer electrophoresis, stained with GelStar (Lonza, Basel, Switzerland) and visualized under ultraviolet trans-illumination. Post-PCR clean-up was performed using EXO-SAP IT (Affymetrix, Foster, CA, USA) according to the manufacturer’s protocol. Sanger sequencing was performed using the BigDye Terminator v1.1 Cycle Sequencing Kit (Applied Biosystems, Foster, CA, USA) on an ABI 3500 genetic analyzer. The sequence trace files obtained were compared with reference sequences NM_018283.2 (*NUDT15*) and NM_000367.4 (*TPMT*) using Mutation Surveyor version 4.0.9 (SoftGenetics, State College, PA, USA) with additional manual verification to detect the target variants. 

### 2.3. Real-Time PCR-HRM Analysis

Genotyping by real-time PCR-HRM analysis was performed on a LightCycler 480 Real-Time PCR System (Roche, Basel, Switzerland). Each reaction contained 10 ng of DNA template, 0.2 µM of each of the primers PCP-0031 (5′-GACCAGCTTTTCTGGGGACT-3′) and PCP-0032 (5′-TCCCACCAGATGGTTCAGAT-3′) for *NUDT15* c.415C>T or PCP-0033 (5′-TTGGGGAATTGACTGTCTTTTT-3′) and PCP-0034 (5′-CATCCATTACATTTTCAGGCTTT-3′) for TPMT*3C, 10 µL of LightCycler LC480 High Resolution Melting Master Mix (Roche, Basel, Switzerland), 3.0 mM MgCl_2_ and nuclease-free water added up to a final volume of 20 µL. Real-time PCR was performed using a step-down PCR protocol as follows: initial denaturation 95 °C for 10 min, subsequent denaturation at 95 °C for 10 s, annealing for 15 s with 0.5 °C decrement per cycle, from 65 to 55 °C, extension at 72 °C for 10 s, for 40 cycles. For post-amplification HRM analysis, the PCR product was denatured at 95 °C for 1 min, renatured at 40 °C for 1 min and subjected to a final melting step from 65 to 95 °C (1 °C per second) with 25 acquisitions per second. Real-time PCR was repeated for samples with a crossing-point (Cp) cycle value higher than 28, optionally with an additional round of DNA purification using the QIAamp kit, to exclude poor amplification due to inadequate template DNA or the presence of inhibitors. The HRM curve analysis was performed using LightCycler 480 Gene Scanning Software using the default normalization for each run and a level 1 temperature-shifting threshold for both targets (Roche, Basel, Switzerland).

### 2.4. PCR-RFLP Analysis

PCR-RFLP genotyping for the two loci was performed as follows. The target region of the *NUDT15* and *TPMT* genes was amplified by PCR using the primers PCP-0023/24 and PCP-0027/28 with the stepdown PCR protocol as described above. Each *NUDT15* c.415C>T genotyping restriction digestion reaction contained 1.7 µL unpurified PCR product, 0.2 µL FastDigest Taal restriction enzyme (Thermo Fisher, Waltham, MA, USA), 0.3 µL 10× FastDigest Green Buffer (Thermo Fisher, Waltham, MA, USA) and nuclease-free water added up to 5 µL. Each TPMT*3C genotyping restriction digestion reaction contained 1.7 µL unpurified PCR product, 0.2 µL AccI restriction enzyme (New England Biolabs, Ipswich, MA, USA), 0.3 µL CutSmart Buffer (New England Biolabs, Ipswich, MA, USA) and nuclease-free water added up to 5 µL. The digestion mix was incubated either at 65 °C (for TaaI digestion) or 37 °C (for AccI digestion) for 15 min on a thermal block (Eppendorf, Hamburg, Germany), and separated alongside 2.0 µL of undigested PCR product using a 2% agarose gel in 1× TBE buffer by electrophoresis at 100 V for 45 min.

## 3. Results

### 3.1. Allele Frequency of Variants and Genetic Prevalence of Thiopurine Intolerance

By DNA sequencing, out of the 60 patients tested, seven (11.7%) were found to be heterozygous and one (1.7%) was found to be homozygous for the *NUDT15*:NM_018283.2:c.415C>T variant at the site (rs116855232). The allele frequency for the variant T allele in this patient cohort was 7.5% (9/120). From previous studies, patients carrying one or more of the loss-of-function missense variants were found to have increased risk of developing leukopenia during thiopurine therapy [26]; thus, the eight patients (13.3%) with one or more mutant alleles were identified to have thiopurine intolerance by *NUDT15* genotyping. The remaining 52 (86.7%) were homozygous for the wild-type C allele and were unaffected.

For the *TPMT*: NM_000367.4:c.719A>G (TPMT*3C) variant site (rs1142345), 59 (98.3%) patients were found to be homozygous for the wild-type allele (*1/*1), one (1.7%) was found to be heterozygous (*3C/*1) and none were found to be homozygous for the variant (*3C/*3C); the corresponding variant allele frequency was 0.83% (1/120) (Table 1). Based on the 2011 and 2013 Clinical Pharmacogenetics Implementation Consortium (CPIC) guidelines [6,8], individuals with one TPMT*3C allele and one *1 allele are classified as “intermediate metabolizer(s)” and individuals with two TPMT*3C alleles are classified as “poor metabolizer(s)”; thus the heterozygous (*3C/*1) patient identified in the current study showed genotypic evidence of reduced thiopurine tolerance by *TPMT* genotyping and the corresponding genetic prevalence in this cohort is 1.7% (1/60). Although phenotyping by TPMT enzyme activity assay was not performed in this study, the genotype-phenotype concordance had been previously demonstrated to be as high as 97% [27], justifying the CPIC guidelines and the classification hereby adopted [8].

There was no patient found to harbor both *NUDT15*:c.415C>T and TPMT*3C variants. The total number of patients found to be at risk of thiopurine intolerance, by genotyping at both loci, was nine out of 60 (15%).

### 3.2. Performance of Real-Time PCR-HRM Genotyping

From Sanger sequencing genotyping, it was found that there was only one sample homozygous for the *NUDT15*:c.415C>T variant and no samples homozygous for the TPMT*3C variant. “Auto Group” analysis, which could assign up to six variant groups, was performed on LightCycler 480 Gene Scanning Software to avoid the inherent bias that may be introduced by performing HRM genotyping on the melting standard sample. 

**Figure 1:**
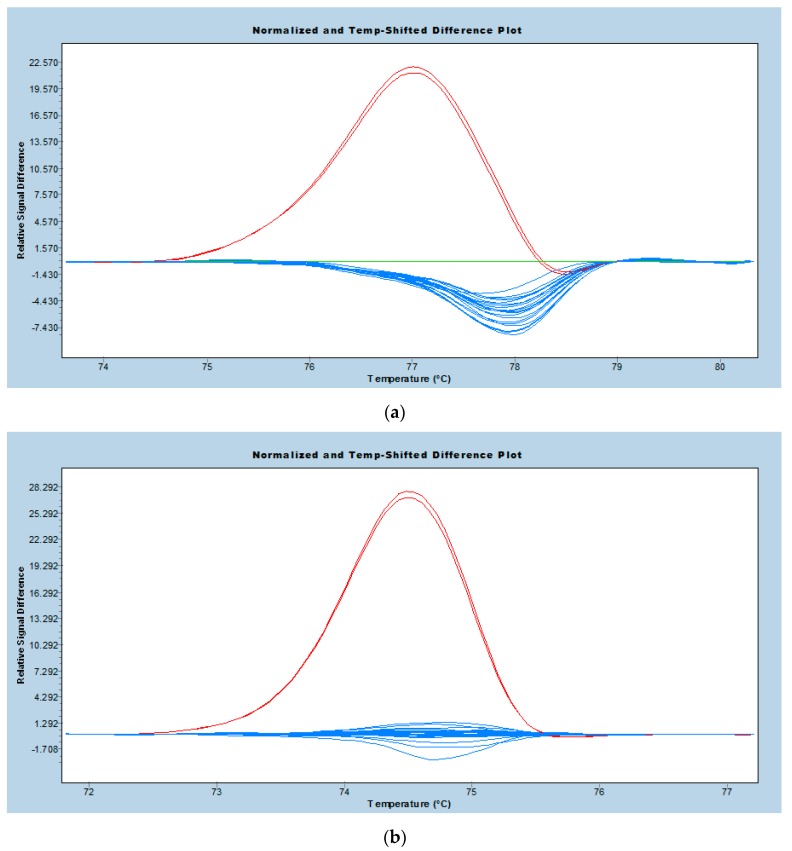
Real-time PCR-high resolution melt (PCR-HRM) difference plots showing: (**a**) heterozygous C/T (red), homozygous T/T (green) and homozygous C/C (blue) samples for *NUDT15* c.415C>T from a representative run; (**b**) heterozygous A/G (red) and homozygous A/A (blue) samples for *TPMT* c.719A>G (TPMT*3C), with the heterozygous sample repeated as a technical replicate.

Under this unsupervised automated grouping analysis, the samples were separated into three groups of 52, seven and one for *NUDT15*:c.415C>T (Figure 1a) and two groups of 59 and one for TPMT*3C (Figure 1b). Inspection of the grouping results showed 100% concordance with the genotyping result from Sanger sequencing (Table S1). 

### 3.3. Performance of PCR-RFLP Genotyping

**Figure 2:**
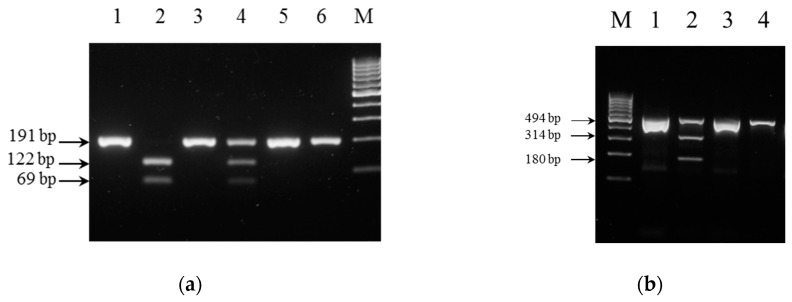
PCR-Restriction Fragment Length Polymorphism (PCR-RFLP) genotyping: (**a**) *NUDT15* genotyping—lanes 1, 3 and 5: undigested samples; lane 2: sample homozygous for *NUDT15* c.415C>T; lane 4: heterozygous sample; lane 6: homozygous wildtype C/C sample, showing no digestion; lane M: 100 bp DNA ladder (Thermo Fisher #SM0242) (**b**) *TPMT* genotyping—lanes 1 and 3: undigested samples; lane 2: sample heterozygous for *TPMT* c.719A>G; lane 4: homozygous wildtype A/A sample, showing no digestion.

The 191 bp PCR product from the wildtype *NUDT15* samples remained after the digestion, whereas all heterozygous *NUDT15*:c.415C>T samples showed additional bands at about 122 and 69 bp after the digestion. Only 122 and 69 bp bands were seen after digestion of the PCR product from the homozygous *NUDT15* c.415C>T sample, showing complete digestion of the 191 bp PCR product (Figure 2a). Similarly, the 494 bp product from the wildtype *TPMT* samples remained after the digestion, whereas the only heterozygous sample found in the study showed additional bands at about 314 and 180 bp, corresponding to predicted digestion products of the amplicon from the *3C allele (Figure 2b). The PCR-RELP genotyping results were also in complete concordance with the genotyping by Sanger sequencing.

## 4. Discussion

Traditional Sanger sequencing has been regarded as the “gold standard” in determining sequence variations, particularly for single nucleotide polymorphisms (SNPs) [28,29]. In many clinical applications, this approach remains valid and preferred as the exact sequence variation may be unknown, or because the discovery and characterization of novel variants is of diagnostic importance. In the context of targeted pharmacogenetic testing, however, targeted genotyping for variants with clear genotype-phenotype correlation is often more useful in guiding dosing optimization and treatment regime.

In this study, two alternative approaches were developed for the genotyping of *NUDT15* c.415C>T and *TPMT* c.719A>G (TPMT*3C) variants. As both variants are base transitions (Class I SNPs), the variant amplicons exhibit relatively large melting temperature shifts and are therefore particularly suited for analysis by real-time PCR-HRM [30]. However, since the effect of both variants have been found to be dose-dependent [31], with individuals suffering most severely from the adverse effect of thiopurines if they carry two copies of the variant alleles [31,32], the consequence of missing a case homozygous for the mutant allele is considerably greater and can be fatal. PCR-RFLP genotyping, therefore, provided a complementary diagnostic aid with particular sensitivity in detecting homozygotes (incomplete restriction digestions, i.e., heterozygotes in both cases, would be more sensitively detected by the HRM analysis). It is doubtful, therefore, whether assays such as allele-specific PCR with no internal control may offer a similarly reliable performance [33]. In fact, although not found in the current study, discrepancies between real-time PCR-based and RFLP-based assays have been reported in other studies [34]. 

Comparing the three techniques (Table 2), Sanger sequencing remains the standard reference method due to its ability to detect non-canonical mutations (including single-nucleotide polymorphisms and small insertions or deletions), accuracy and relative robustness to input DNA quantity and quality. While the recommended amount of template DNA, according to the manufacturer (Available online: http://tools.thermofisher.com/content/sfs/manuals/cms_061443.pdf), is 10^4^ copies (~34.5 ng of human genomic DNA) or above, evaluation using the PCR protocol described in this study achieved a limit of detection (LOD) of ≤1.25 ng of human genomic DNA (Methods S2). Further optimizations, including increasing the number of PCR cycles [35], may bring the LOD down to the theoretical limit of a single genomic copy [36]; nevertheless, as a clinical laboratory performing diagnostic testing, it may be preferable to balance among economical use of template DNA, reasonable number of PCR cycles and standardizing laboratory protocols to avoid excessive pipetting and manual dilution steps. With regard to these, PCR-HRM is considered less robust compared to Sanger sequencing and PCR-RFLP, which depend only on the presence of adequate amplicon for subsequent cycle sequencing and restriction enzyme digestion, rather than comparable amplification efficiency within a critical number of cycles [37].

While the robustness and versatility of Sanger sequencing genotyping are evident in the application to *NUDT15* c.415C>T and TPMT*3C, it should perhaps be noted that PCR-HRM and PCR-RFLP each have a reagent cost less than one-third of sequencing genotyping, and have a turnaround time about one-third to one-fourth of that of sequencing, even if the whole Sanger sequencing process is streamlined (Table 2). Furthermore, PCR-HRM and PCR-RFLP genotyping do not require manual inspection of sequencing traces and their interpretation can be considered relatively straightforward. Depending on local reagent and labor costs, the actual increase in cost-effectiveness and efficiency may vary, but it is expected that adoption of either or both of the alternate genotyping methods will generally lead to reagent cost saving and reduced hands-on/turnaround time compared to the Sanger sequencing workflow [38]. 

A limitation of the current study is its relatively small sample size (*n* = 60) which precluded extensive validation of the methods. From the Exome Aggregation Consortium (ExAC) database (Available online: http://exac.broadinstitute.org/), the East Asian subpopulation allele frequency for *NUDT15* c.415C>T is 0.1043 and that for TPMT*3C is 0.01319. Calculations under Hardy-Weinberg assumptions predict that, for every 60 East Asian individuals, there would be 48.3 homozygous for the *NUDT15* wildtype allele, 11.0 heterozygotes and 0.72 homozygous for the *NUDT15* c.415C>T variant; for the TPMT*3C variant, the respective numbers would be 58.4, 1.56 and 0.01. As can be seen from the results (Table 1), the data from the ExAC database fell within the 95% confidence interval of the point estimates of the allele frequencies, as may be expected. Assuming our patient population presenting for genetic testing other than for *NUDT15* and *TPMT* genotyping do not have increased prevalence of the *NUDT15* c.415C>T and TPMT*3C variants compared to the ExAC East Asian subpopulation, it would require genotyping a few hundred (for *NUDT15* c.415C>T) to tens of thousands (for TPMT*3C) of patients to collect even a few samples for establishing homozygous mutant genotype samples for HRM analysis. Due to facility and resource limitations, this approach was not attempted; in actual practice, this warrants the simultaneous use of PCR-RFLP to prevent false negatives.

Prospective studies, with additional blood samples taken for haematological, on enzyme activity and thiopurine metabolite analysis may reveal more complex interactions between the susceptibility of alleles *NUDT15* c.415C>T and TPMT*3C. However, since only archived DNA samples from patients receiving genetic testing for unrelated conditions were used, and none of them were reported to be administered thiopurines, a breakdown of the adverse drug reactions according to genotyping and predicted metabolizer status could not be performed.

Further genotyping studies may establish synthetic oligonucleotide targets as controls for the HRM or RFLP analysis, given the relatively small PCR amplicon. Of note, however, as from our in-house evaluation, real-time PCR-HRM genotyping is relatively susceptible to quantitative changes in input DNA amount, even when the analysis is run in the presence of the known genotype synthetic sample, the validity of the HRM analysis may still not be guaranteed. A potentially feasible approach would be spiking and/or pooling: as spiking of HRM mixture by wildtype (“control”) DNA may enhance the detection of the rare homozygous mutants [39], it may be used to enhance the reliability of the HRM assay shall ambiguous genotypes arise; moreover, as HRM analysis can potentially detect a “mutant load” down to 3–5% [40], pooling of up to 20 samples may be possible for time and cost-efficient screening studies of TPMT*3C variants in East Asian or Chinese populations.

## 5. Conclusions

The complementary real-time PCR-HRM and PCR-RFLP genotyping methods were successfully developed as alternatives to traditional Sanger sequencing for the cost-effective detection of *NUDT15* c.415C>T and *TPMT* c.719A>G (TPMT*3C) variants. Further prospective studies may establish their roles in the genotype-informed dosage adjustment of thiopurines, the use of alternative agents in selected patient subgroups and the prevention of morbidity and mortality due to thiopurine intolerance.

## Figures and Tables

**Table 1:** Genotypes at the *NUDT15* c.415C>T and TPMT*3C thiopurine-intolerant variant sites.

Gene Target (dbSNP ID)	Homozygous Wildtype ^1^	Heterozygous	Homozygous Mutant	Minor Allele Frequency (95% Confidence Interval) ^2^
*NUDT15* c.415C>T (rs116855232)	52 (86.7%)	7 (11.7%)	1 (1.7%)	0.075 (0.037–0.14) ^3^
*TPMT* c.719A>G (rs1142345)	59 (98.3%)	1 (1.7%)	0 (0.0%)	0.0083 (0.00044–0.052) ^4^

^1^ The wildtype allele is defined as base C for *NUDT15* c.415 and A for *TPMT* c.719, respectively; ^2^ Calculated using one-sample proportions test with continuity correction using R version 3.3.1; ^3^ The Exome Aggregation Consortium (ExAC) East Asian minor allele (T) frequency is 0.1043; ^4^ The ExAC East Asian minor allele (G) frequency is 0.01319.

**Table 2:** Comparison of three genotyping approaches for *NUDT15* c.415C>T and *TPMT* c.719A>G (TPMT*3C) variants.

Method	Percentage of Mutants Detected ^1^	Minimum Amount of Input DNA (ng) ^2^	Cost per Sample (USD) ^3^	Turn-around Time (h) ^4^	Interpretation	Capability to Detect Non-Canonical Mutations
Sanger sequencing	Reference method	≤1.25	16	7.5 or 1 working day	Simple, but may be automated and enhanced by software	Yes
PCR-high resolution melt (PCR-HRM)	*NUDT15* c.415C>T: 100% (8/8) TPMT*3C: 100% (1/1)	10	5	2	Requires special software package provided by realtime PCR platform vendor	Limited ^5^
PCR-restriction fragment length polymorphism (PCR-RFLP)	*NUDT15* c.415C>T: 100% (8/8) TPMT*3C: 100% (1/1)	≤1.25	4	3	Very simple, by comparing band patterns on gel	No

^1^ Including both heterozygous and homozygous mutant samples; ^2^ The values of minimum amount of input DNA came from additional evaluation (Table S2). Actual amounts used in the assays, except for PCR-HRM, were chosen for robust amplification performance across a range of in-house genetic tests routinely performed, including, but not limited to multiplex PCR; ^3^ Cost estimations based on calculations by Fateh et al. [38] (see text) for Sanger sequencing and PCR-RFLP. The cost of PCR-HRM was estimated using quotes obtained from the local reagent vendor and included the PCR consumables used for the experiment; ^4^ Excluding time needed for genomic DNA isolation. Minimum turn-around time of Sanger sequencing genotyping was based on: 2 h for first PCR, 1 h for gel electrophoresis, 0.5 h for PCR product purification, 2 h for the cycle sequencing reaction and finally 2 h for capillary electrophoresis on the sequencer. The HRM runtime was less than 1.5 h and the restriction enzyme digestion took 15 min; ^5^ PCR-HRM analysis can additionally detect non-canonical variants, e.g., *TPMT* c.719A>C, although assay sensitivity and specificity will depend on the specific mutation involved, DNA amount and quality, and the presence of additional sequence alterations in the genotyped region.

## Supplementary Materials

The following are available online at www.mdpi.com/2075-4418/07/2/27/s1.

## Abbreviations

TPMT	Thiopurine *S*-methyltransferase
NUDT15	Nudix hydrolase 15
