# Supplementary material for: Comparison of Direct Sequencing, Real-Time PCR-High Resolution Melt (PCR-HRM) and PCR-Restriction Fragment Length Polymorphism (PCR-RFLP) Analysis for Genotyping of Common Thiopurine Intolerant Variant Alleles NUDT15 c.415C>T and TPMT c.719A>G (TPMT*3C)
Source: Diagnostics (Basel). 2017 May 12;7(2):27. doi: 10.3390/diagnostics7020027 (PMC5489947; doi:10.3390/diagnostics7020027)
Supplement: Supplementary file 1 [file diagnostics-07-00027-s001.zip › diagnostics-194498 supplementary resubmit/Methods S2.docx]

**Supplementary Methods S2**: Evaluation of the PCR protocol using low amounts of input DNA

To test whether the PCR protocol is compatible with lower amount of input DNA, PCR was performed with dilutions and decreasing amount of input DNA from 20 ng down to 1.25 ng (a 16-fold range). Further dilutions were not performed.

As poor PCR product yield is usually self-evident on Sanger sequencing rather than presenting as false positives and negatives, only restriction enzyme digestion was performed for the lowest input PCR reactions (2.5 ng, 1.25 ng). Heterozygous samples were chosen for this evaluation as false results can be caused by inability to detect any of the smaller, digested product bands.

| **Genomic DNA input (ng)** | **Equivalent human genomic copies^1^** | **PCR product for *NUDT15* c.415C>T genotyping** | **PCR product for *TPMT**3C genotyping** | **Digested**  **PCR product for *NUDT15* c.415C>T genotyping**  **(size, bp)** | **Digested**  **PCR product for *TPMT**3C genotyping**  **(size, bp)** |
| --- | --- | --- | --- | --- | --- |
| 20 | 5800 | ✔ | ✔ | Not done | Not done |
| 15 | 4350 | ✔ | ✔ | Not done | Not done |
| 10 | 2900 | ✔ | ✔ | Not done | Not done |
| 5.0 | 1450 | ✔ | ✔ | Not done | Not done |
| 2.5 | 725 | ✔ | ✔ | ✔  191, 122, 69 | ✔  494, 314, 180 |
| 1.25 | 363 | ✔ | ✔ | ✔  191, 122, 69 | ✔  494, 314, 180 |
| 0 | 0 | × | × | Not done | Not done |

^1^ Number of equivalent haploid human genomic DNA copies calculated using the ThermoFisher DNA Copy Number and Dilution Calculator, freely available at <https://www.thermofisher.com/hk/en/home/brands/thermo-scientific/molecular-biology/molecular-biology-learning-center/molecular-biology-resource-library/thermo-scientific-web-tools/dna-copy-number-calculator.html>

| 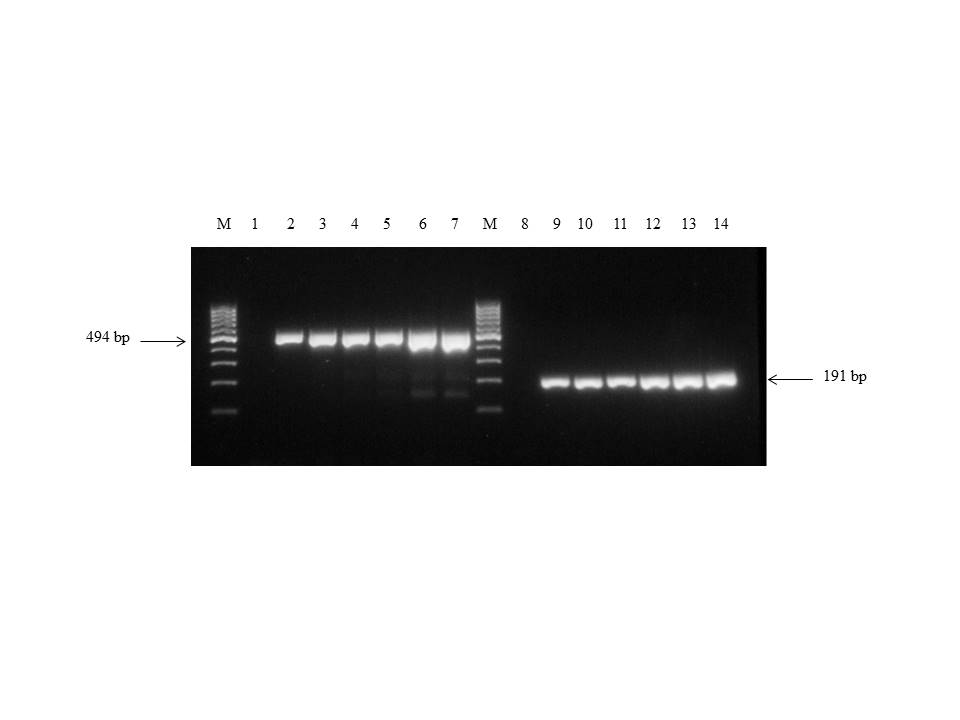  (**a**) |
| --- |
| 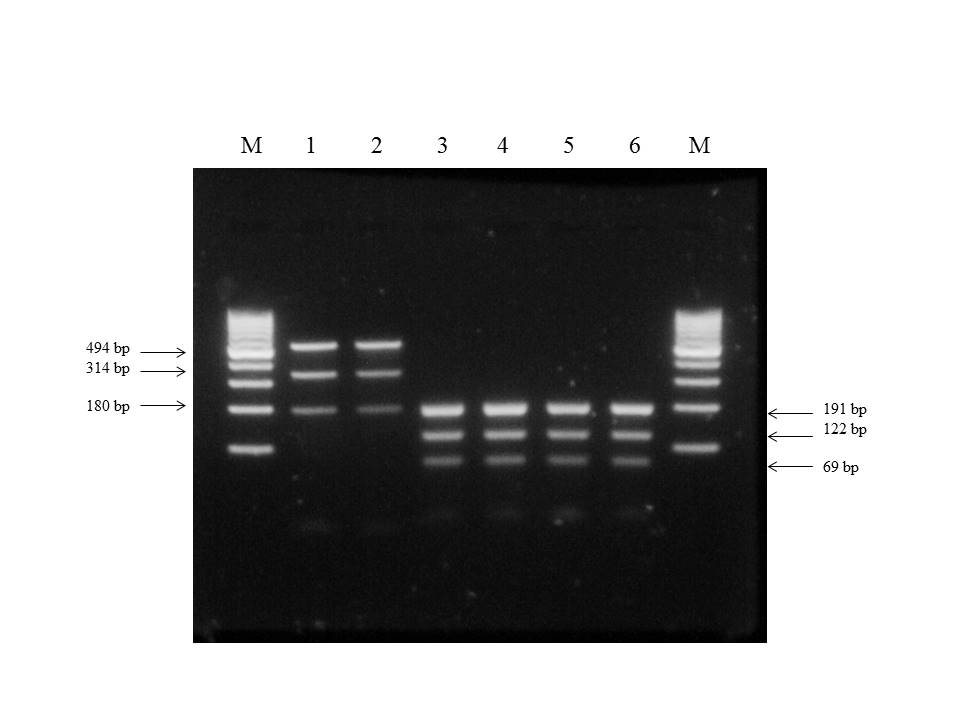  (b) |

**Supplementary Methods S2, Figure 1.** Effect of decreasing amount of input DNA: (**a**) PCR products for *TPMT* c.719A>G (*TPMT**3C) (lane 1-7) and *NUDT15* c.415C>T (lane 8-14) genotyping; lane 1 and 8: 0 ng genomic DNA sample input; lane 2 and 9: 1.25 ng genomic DNA sample input; lane 3 and 10: 2.5 ng genomic DNA sample input; lane 4 and 11: 5 ng genomic DNA sample input; lane 5 and 12: 10 ng genomic DNA sample input; lane 6 and 13: 15 ng genomic DNA sample input; lane 7 and 14: 20 ng genomic DNA sample input; ; lane M: 100 bp DNA ladder (ThermoFisher #SM0242) (**b**) digested PCR products for *TPMT* c.719A>G lane 1 -2 and *NUDT15* genotyping c.415C>T lane 3-6; lane 1 digested PCR products from 1.25 ng genomic DNA sample input; lane 2 digested PCR products from 2.5 ng genomic DNA sample input; lane 3 and 5 digested PCR products from 1.25 ng genomic DNA sample input; lane 4 and 6 digested PCR products from 2.5 ng genomic DNA sample input; lane M: 100 bp DNA ladder (Thermo Fisher #SM0242)
