# Supplementary material for: Comparison of Direct Sequencing, Real-Time PCR-High Resolution Melt (PCR-HRM) and PCR-Restriction Fragment Length Polymorphism (PCR-RFLP) Analysis for Genotyping of Common Thiopurine Intolerant Variant Alleles NUDT15 c.415C>T and TPMT c.719A>G (TPMT*3C)
Source: Diagnostics (Basel). 2017 May 12;7(2):27. doi: 10.3390/diagnostics7020027 (PMC5489947; doi:10.3390/diagnostics7020027)
Supplement: Supplementary file 1 [file diagnostics-07-00027-s001.zip › diagnostics-194498 supplementary resubmit/Table S1.docx]

**Table S1**: Genotyping result of all 60 samples by sequencing, real-time PCR-HRM and PCR-RFLP analysis

1. For *NUDT15* c.415C>T, genotypes are designated as wild type (C/C), heterozygous (C/T) and homozygous mutant (T/T).
2. For *TPMT* c.719A>G (*TPMT**3C), genotypes are designated as *1/*1 (wild type, A/A), *1/*3C (heterozygous, A/G) and *3C/*3C (homozygous mutant, G/G). Note that *3C/*3C was not identified in this study.

|  | *NUDT15* c.415C>T genotyping | | | | | *TPMT**3C genotyping | | | | |
| --- | --- | --- | --- | --- | --- | --- | --- | --- | --- | --- |
| **SAMPLE ID** | PCR-RFLP digested  PCR product size (base pair) | | | Sanger sequencing results | High resolution melting results  (Auto group) | PCR-RFLP digested  PCR product size (base pair) | | | Sanger sequencing results | High resolution melting results  (Auto group) |
|  | 191 | 122 | 69 |  |  | 494 | 314 | 180 |  |  |
| **1** | ✔ | ✔ | ✔ | **c. 415C>T**  **Heterozygous** | 1 | ✔ |  |  | *1/*1 | 2 |
| **2** | ✔ |  |  | Wild Type | 2 | ✔ |  |  | *1/*1 | 2 |
| **3** | ✔ |  |  | Wild Type | 2 | ✔ |  |  | *1/*1 | 2 |
| **4** | ✔ |  |  | Wild Type | 2 | ✔ |  |  | *1/*1 | 2 |
| **5** | ✔ |  |  | Wild Type | 2 | ✔ |  |  | *1/*1 | 2 |
| **6** | ✔ |  |  | Wild Type | 2 | ✔ |  |  | *1/*1 | 2 |
| **7** | ✔ |  |  | Wild Type | 2 | ✔ |  |  | *1/*1 | 2 |
| **8** | ✔ | ✔ | ✔ | **c. 415C>T**  **Heterozygous** | 1 | ✔ |  |  | *1/*1 | 2 |
| **9** | ✔ |  |  | Wild Type | 2 | ✔ |  |  | *1/*1 | 2 |
| **10** | ✔ |  |  | Wild Type | 2 | ✔ |  |  | *1/*1 | 2 |
| **11** | ✔ |  |  | Wild Type | 2 | ✔ |  |  | *1/*1 | 2 |
| **12** | ✔ |  |  | Wild Type | 2 | ✔ |  |  | *1/*1 | 2 |
| **13** | ✔ |  |  | Wild Type | 2 | ✔ |  |  | *1/*1 | 2 |
| **14** | ✔ |  |  | Wild Type | 2 | ✔ |  |  | *1/*1 | 2 |
| **15** | ✔ |  |  | Wild Type | 2 | ✔ |  |  | *1/*1 | 2 |
| **16** | ✔ |  |  | Wild Type | 2 | ✔ |  |  | *1/*1 | 2 |
| **17** | ✔ | ✔ | ✔ | **c. 415C>T**  **Heterozygous** | 1 | ✔ |  |  | *1/*1 | 2 |
| **18** | ✔ |  |  | Wild Type | 2 | ✔ |  |  | *1/*1 | 2 |
| **19** | ✔ |  |  | Wild Type | 2 | ✔ |  |  | *1/*1 | 2 |
| **20** | ✔ |  |  | Wild Type | 2 | ✔ |  |  | *1/*1 | 2 |
| **21** | ✔ |  |  | Wild Type | 2 | ✔ |  |  | *1/*1 | 2 |
| **22** | ✔ |  |  | Wild Type | 2 | ✔ |  |  | *1/*1 | 2 |
| **23** | ✔ |  |  | Wild Type | 2 | ✔ |  |  | *1/*1 | 2 |
| **24** | ✔ |  |  | Wild Type | 2 | ✔ |  |  | *1/*1 | 2 |
| **25** | ✔ |  |  | Wild Type | 2 | ✔ |  |  | *1/*1 | 2 |
| **26** | ✔ |  |  | Wild Type | 2 | ✔ |  |  | *1/*1 | 2 |
| **27** | ✔ |  |  | Wild Type | 2 | ✔ |  |  | *1/*1 | 2 |
| **28** | ✔ |  |  | Wild Type | 2 | ✔ |  |  | *1/*1 | 2 |
| **29** | ✔ |  |  | Wild Type | 2 | ✔ |  |  | *1/*1 | 2 |
| **30** | ✔ |  |  | Wild Type | 2 | ✔ |  |  | *1/*1 | 2 |
| **31** | ✔ |  |  | Wild Type | 2 | ✔ |  |  | *1/*1 | 2 |
| **32** | ✔ |  |  | Wild Type | 2 | ✔ |  |  | *1/*1 | 2 |
| **33** | ✔ |  |  | Wild Type | 2 | ✔ |  |  | *1/*1 | 2 |
| **34** | ✔ |  |  | Wild Type | 2 | ✔ |  |  | *1/*1 | 2 |
| **35** | ✔ |  |  | Wild Type | 2 | ✔ |  |  | *1/*1 | 2 |
| **36** | ✔ |  |  | Wild Type | 2 | ✔ |  |  | *1/*1 | 2 |
| **37** | ✔ | ✔ | ✔ | **c. 415C>T**  **Heterozygous** | 1 | ✔ |  |  | *1/*1 | 2 |
| **38** | ✔ |  |  | Wild Type | 2 | ✔ |  |  | *1/*1 | 2 |
| **39** | ✔ | ✔ | ✔ | **c. 415C>T**  **Heterozygous** | 1 | ✔ |  |  | *1/*1 | 2 |
| **40** | ✔ |  |  | Wild Type | 2 | ✔ |  |  | *1/*1 | 2 |
| **41** | ✔ |  |  | Wild Type | 2 | ✔ |  |  | *1/*1 | 2 |
| **42** | ✔ |  |  | Wild Type | 2 | ✔ |  |  | *1/*1 | 2 |
| **43** | ✔ |  |  | Wild Type | 2 | ✔ |  |  | *1/*1 | 2 |
| **44** | ✔ |  |  | Wild Type | 2 | ✔ |  |  | *1/*1 | 2 |
| **45** | ✔ |  |  | Wild Type | 2 | ✔ |  |  | *1/*1 | 2 |
| **46** | ✔ |  |  | Wild Type | 2 | ✔ | ✔ | ✔ | ***1/*3C** | 1 |
| **47** | ✔ |  |  | Wild Type | 2 | ✔ |  |  | *1/*1 | 2 |
| **48** | ✔ |  |  | Wild Type | 2 | ✔ |  |  | *1/*1 | 2 |
| **49** | ✔ |  |  | Wild Type | 2 | ✔ |  |  | *1/*1 | 2 |
| **50** | ✔ |  |  | Wild Type | 2 | ✔ |  |  | *1/*1 | 2 |
| **51** | ✔ |  |  | Wild Type | 2 | ✔ |  |  | *1/*1 | 2 |
| **52** | ✔ |  |  | Wild Type | 2 | ✔ |  |  | *1/*1 | 2 |
| **53** | ✔ |  |  | Wild Type | 2 | ✔ |  |  | *1/*1 | 2 |
| **54** | ✔ | ✔ | ✔ | **c. 415C>T**  **Heterozygous** | 1 | ✔ |  |  | *1/*1 | 2 |
| **55** | ✔ | ✔ | ✔ | **c. 415C>T**  **Heterozygous** | 1 | ✔ |  |  | *1/*1 | 2 |
| **56** |  | ✔ | ✔ | **c. 415C>T**  **Homozygous** | 3 | ✔ |  |  | *1/*1 | 2 |
| **57** | ✔ |  |  | Wild Type | 2 | ✔ |  |  | *1/*1 | 2 |
| **58** | ✔ |  |  | Wild Type | 2 | ✔ |  |  | *1/*1 | 2 |
| **59** | ✔ |  |  | Wild Type | 2 | ✔ |  |  | *1/*1 | 2 |
| **60** | ✔ |  |  | Wild Type | 2 | ✔ |  |  | *1/*1 | 2 |
